# Supplementary material for: Ventrointermediate thalamic stimulation improves motor learning in humans
Source: Commun Biol. 2024 Jul 2;7:798. doi: 10.1038/s42003-024-06462-5 (PMC11220095; doi:10.1038/s42003-024-06462-5)
Supplement: Supplementary file 2 — Supplementary Material [file 42003_2024_6462_MOESM2_ESM.pdf]

Supplementary material

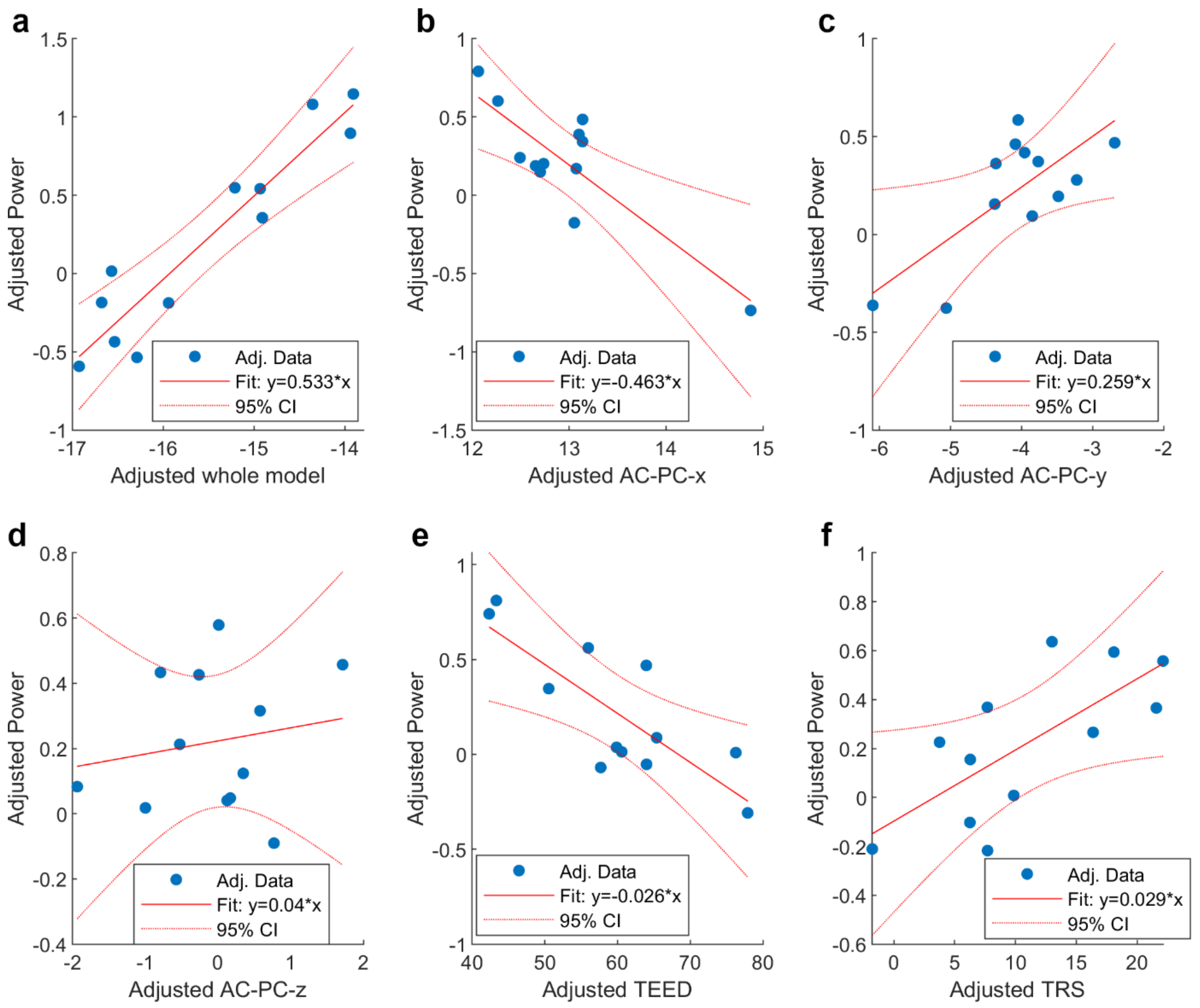

Supplementary Figure 1 Visualization of the multiple linear regression analysis. **a** Multiple linear regression plot, visualizing the fit of all the independent variables (electrode locations in relation to the anterior and posterior commissures (AC–PC line), lateral to the midline (x), posterior to the mid-commissural point (y), and inferior to the inter-commissural plane (z), the total electrical energy delivered (TEED), and the tremor rating score (TRS)) against the dependent variable (power). Adjusted R^2: 0.791, p-value = 0.009. **b** Partial regression plot, showing the marginal importance of the AC–PC-x coordinate of the electrode location to the model. **c** Fit of AC–PC-y coordinate of the electrode location against the dependent variable power within the model. **d** Fit of AC–PC-z coordinate of the electrode location against the dependent variable power within the model. **e** Fit of TEED against the dependent variable power within the model. **f** Fit of TRS against the dependent variable power within the model.

Supplementary Table 1 Regression results using mean power with DBS-ON as the criterion (n = 12). Predictor variables were electrode locations in relation to the AC–PC line (mm) lateral to the midline (x), posterior to the mid-commissural point (y), and inferior to the inter-commissural plane (z), the total electrical energy delivered (TEED), and the tremor rating score (TRS), while stimulation was on.

	Unstandardized Coefficients		Standardized Coefficients			95% Confidence Interval	
Model	B	*SE*	Beta	*T*	Significance	LL	UL
Constant	8.494	2.224		3.820	0.009**	3.053	13.935
AC–PC-x (right)	-0.463	0.123	-1.424	-3.776	0.009**	-0.762	-0.163
AC–PC-y (right)	0.259	0.099	0.681	2.615	0.040*	0.017	0.501
AC–PC-z (right)	0.040	0.092	0.122	0.437	0.677	-0.185	0.266
TEED (right)	-0.026	0.008	-0.775	-3.303	0.016*	-0.045	-0.588
TRS-ON	0.029	0.012	0.431	2.491	0.047*	0.001	0.058

Note: [a] Dependent variable: mean power (r = 0.941, r^2 = 0.886, Adj. r^2 = 0.791).
* indicates p < 0.05, ** indicates p < 0.01.

Supplementary Table 2 Patient information and stimulation parameters. Values reported are the tremor rating score (TRS), while stimulation was on, and the stimulation type (m = monopolar; b = bipolar) for the left/right stimulating electrode. Stimulation frequency (Hz) was identical for both hemispheres. Amplitude (V = volts; mA = milliampere), pulse width (µs = microseconds), and electrode location are given separately for the left and right hemisphere. Electrode locations are given in relation to the AC–PC line (mm) lateral to the midline (x), posterior to the mid-commissural point (y), and inferior to the inter-commissural plane (z).

						Left hemisphere					Right hemisphere				
								AC–PC					AC–PC		
ID	Gender	Age	TRS during DBS-ON	Stim Type	Frequency	Amplitude	Pulse width	x	y	z	Amplitude	Pulse width	x	y	z
1	f	79	0	m/m	130	2.4 V	60	-11.5	-7.0	-3.2	2.0 V	60	11.3	-5.0	-2.2
2	m	74	20	m/m	130	2 mA	40	-12.1	-7.5	-3.0	3.5 mA	40	11.0	-4.5	-2.9
3	f	66	5	b/b	130	3.5 V	60	-13.1	-2.5	1.4	3.0 V	60	13.5	-2.5	0.9
4	m	78	30	m/m	130	6.0 mA	20	-14.6	-5.0	0.8	3.5 mA	40	12.1	-6.0	-1.0
5	m	80	12	m/m	130	2.5 V	60	-12.5	-4.0	-2.5	2.5 V	60	17.3	0.0	2.4
6	f	74	15	m/m	130	2.9 mA	60	-15.3	-5.0	0.6	3.0 mA	60	14.6	-3.0	1.8
7	m	73	9	m/m	130	3.25 mA	40	-12.2	-5.5	-2.1	3.0 mA	40	14.9	-4.5	-0.5
8	m	77	13	b/b	130	2.0 V	60	-13.2	-4.5	0.1	2.8 V	60	13.3	-5.5	1.7
9	f	60	0	m/b	130	2.0 V	90	-12.1	-4.0	-0.8	2.5 V	90	10.5	-4.5	-1.0
10	m	78	20	m/m	130	1.8 V	60	-14.7	-4.5	1.4	2.6 V	60	12.9	-5.5	1.5
11	f	62	1	m/m	130	2.8 V	60	-12.4	-3.5	0.1	3.6 V	60	11.8	-3.5	1.3
12	f	67	6	m/m	130	2.6 mA	40	-12.4	-2.5	-1.5	2.6 mA	40	12.2	-4.5	-2.8
